# Capsular polysaccharides of *Acinetobacter baumannii* modulate antimicrobial resistance and innate immune response

**DOI:** 10.1038/s41598-026-44001-w

**Published:** 2026-03-21

**Authors:** Laurita Klimkaite, Giedre Kukanauskaite, Joris Naujalis, Danas Ivanauskas, Dominykas Grigorjevas, Greta Petrenaite, Meda Skinkyte, Jekaterina Porchun, Egle Zalyte, Irina Buchovec, Jurate Skerniskyte

**Affiliations:** 1 Institute of Biosciences, Life Sciences Center, Vilnius University, Vilnius, 10257 Lithuania; 2 Institute of Photonics and Nanotechnology, Faculty of Physics, Vilnius University, Vilnius, 10222 Lithuania

**Keywords:** *Acinetobacter baumannii*, Capsular polysaccharides, Biofilms, PDT, Innate immunity, Apoptosis, Immunology, Microbiology

## Abstract

*Acinetobacter baumannii* is an opportunistic pathogen characterised by multidrug resistance and is among the leading causes of nosocomial infections. This study investigated the role of capsular polysaccharides (CPS) in antimicrobial resistance and host-pathogen interaction. CPS-deficient mutant (∆*galU*) displayed increased susceptibility to gentamicin, tetracycline, colistin, and sodium dodecyl sulfate (SDS). In the absence of CPS, bacteria adapted by forming biofilm, characterised by a thicker extracellular matrix. These biofilms displayed increased resistance to colistin, chlorhexidine, and hydrogen peroxide, but remained sensitive to tetracycline and SDS. CPS were also essential for the resistance to photodynamic therapy induced by blue light and chlorophyllin. Gene expression analysis revealed upregulation of *galU* gene under the exposure to antibiotics, blue light, fetal bovine serum, and during the contact with macrophages. CPS-deficient mutant and its derived outer membrane vesicles elicited a stronger pro-inflammatory response, compared to wild-type. CPS shielding *A. baumannii* demonstrated the ability to induce caspase-3 activation to a greater extent compared to the mutant strain. Moreover, purified CPS induced pro-inflammatory cytokine expression in sterile infection and promoted neutrophil chemotaxis. Altogether, this study demonstrates that CPS not only mask *A. baumannii* virulence surface structures, but also actively modulate host immune response and antimicrobial resistance.

**Supplementary Information:**

The online version contains supplementary material available at 10.1038/s41598-026-44001-w.

## Introduction

Due to increasing bacterial antibiotic resistance, hospital-acquired infections contribute to rising patient mortality in intensive care units (ICUs)^1^. The ESKAPE group of pathogens comprises the most frequent opportunistic bacteria encountered in ICUs, exhibiting extensive multidrug resistance (MDR) and posing a critical threat to patient outcomes and infection control strategies. The World Health Organisation declared the highest priority group of Gram-negative bacteria, including carbapenem-resistant *Acinetobacter baumannii*, *Pseudomonas aeruginosa*, and *Enterobacteriaceae*, which urgently need new treatment strategies^2^.

*A. baumannii* is one of the leading infection agents in ICUs, commonly causing ventilator-associated pneumonia, bloodstream infections, and urinary tract infections^3^. It can persist in hospital environments due to its ability to survive on medical surfaces by forming biofilms, contributing to interinstitutional spread^4^. The surfaces in hospitals are sterilised using various disinfectants. However, bacterial biofilms are highly resistant to antimicrobials^5^. Alcohols are one of the most often used disinfectants: for skin, surfaces, and equipment preparation^6^. Hydrogen peroxide is preferred for high-level disinfection and sterilisation of medical equipment and surfaces. Chlorhexidine-impregnated central line catheters are used to reduce the risk of catheter-related bloodstream infections. Chlorhexidine and sodium dodecyl sulfate (SDS) can be found in medical soaps and disinfecting solutions; they can also be used in hospital-grade detergents to clean surfaces and help remove bacterial biofilms. Carbapenems are the first-line treatment for infections caused by susceptible *A. baumannii*^7^. Carbapenem-resistant *A. baumannii* can be treated with sulbactam/durlobactam-based therapies, polymyxins, cefiderocol, tetracyclines, fluoroquinolones, and aminoglycosides. As resistance to conventional antibiotics grows, alternative therapeutic strategies are being explored, including phage therapy and antimicrobial photodynamic therapy (PDT). PDT combines a non-toxic photosensitizer with visible light to generate reactive oxygen species that inactivate bacterial cells^8^.

Alternative antimicrobial techniques often target the virulence factors produced by *A. baumannii*. These factors are considered critical for the bacterium’s persistence both on medical equipment and in immunocompromised patients. The best characterised virulence factor of *A. baumannii* is the outer membrane protein A (OmpA), which contributes to antibiotic resistance, biofilm formation, resistance to serum, and contributes to inflammation during infection in vivo^9–13^. Additional virulence factors of *A. baumannii* were identified, such as adhesins Bap, Blp1, iron acquisition systems, phospholipases, and glycoprotease CpaA^14–18^. Recent findings demonstrated the importance of outer membrane vesicles in the pathogenesis of *A. baumannii*^19–21^.

*A. baumannii* can detach from biofilms on catheters or intubation tubes and infect patients^22^. In immunosuppressed patients, components of innate immunity play an important role in protection against infectious agents^23,24^. Studies show that innate immunity cells, such as macrophages, monocytes, neutrophils and dendritic cells, mediate bacterial clearance and activate the production of co-stimulatory molecules during *A. baumannii* infection^25,26^. Delayed neutrophil recruitment and increased IL-1β/IL-18 production were observed to be dependent on the NLRP3-ASC-caspase-1/caspase-11 pathway in a mouse pneumonia model, highlighting the importance of inflammasome formation in the clearance of *A. baumannii* infection^27^. OmpA protein was able to induce autophagy in bone marrow-derived dendritic cells involving the PI3K/mTOR pathway^28^. OmpA was also found in mitochondria and induced apoptosis^29^. Omp33-36 porin induced apoptosis by blocking autophagy in human cells^30^. However, very little is still known about the host’s innate immune response to virulence factors of *A. baumannii*.

*A. baumannii* produces capsular polysaccharides (CPS) via the Wzy/Wzx-dependent system, which protects the bacterium against desiccation, antimicrobials, ascitic fluid, and phagocytosis^31,32^. CPS production is mediated by genes located within the K locus. *A. baumannii* is characterised by high variability in its capsule locus, with more than 200 distinct K loci identified^33^. It has been shown that *A. baumannii* can regulate the expression of K locus genes under antibiotic stress and in human serum^34–37^. Despite increasing evidence of its importance, the specific role of CPS in *A. baumannii* pathogenesis has not yet been fully elucidated.

Here, we aimed to demonstrate the range of virulence traits determined by CPS produced by *A. baumannii*. We investigated two aspects: the ability of *A. baumannii* to survive outside the host under stress conditions potentially encountered in the hospital environment, and its interaction with the components of innate immunity. We compared the virulence traits of the clinical *A. baumannii* strain and its CPS non-producing ∆*galU*-deletion mutant. Our findings demonstrated that CPS can protect *A. baumannii* from antimicrobials and photodynamic treatment-induced stresses, although the importance of CPS is not uniform across all tested conditions. The expression of *galU* gene was induced by the presence of both antimicrobials and components of innate immunity. Furthermore, the findings revealed that during *A. baumannii* infection, CPS reduced the activation of pro-inflammatory responses while promoting the activation of apoptosis-associated caspases, thereby contributing to the suppression of innate immune responses. However, under sterile conditions, purified CPS exhibited pro-inflammatory properties and induced neutrophil migration. Our study highlights CPS as one of the key virulence factors of *A. baumannii*, contributing to the protection against both antimicrobials and components of the innate immunity.

## Results

### The presence of CPS modulates *A. baumannii* resistance to antimicrobial agents

**Fig. 1:**
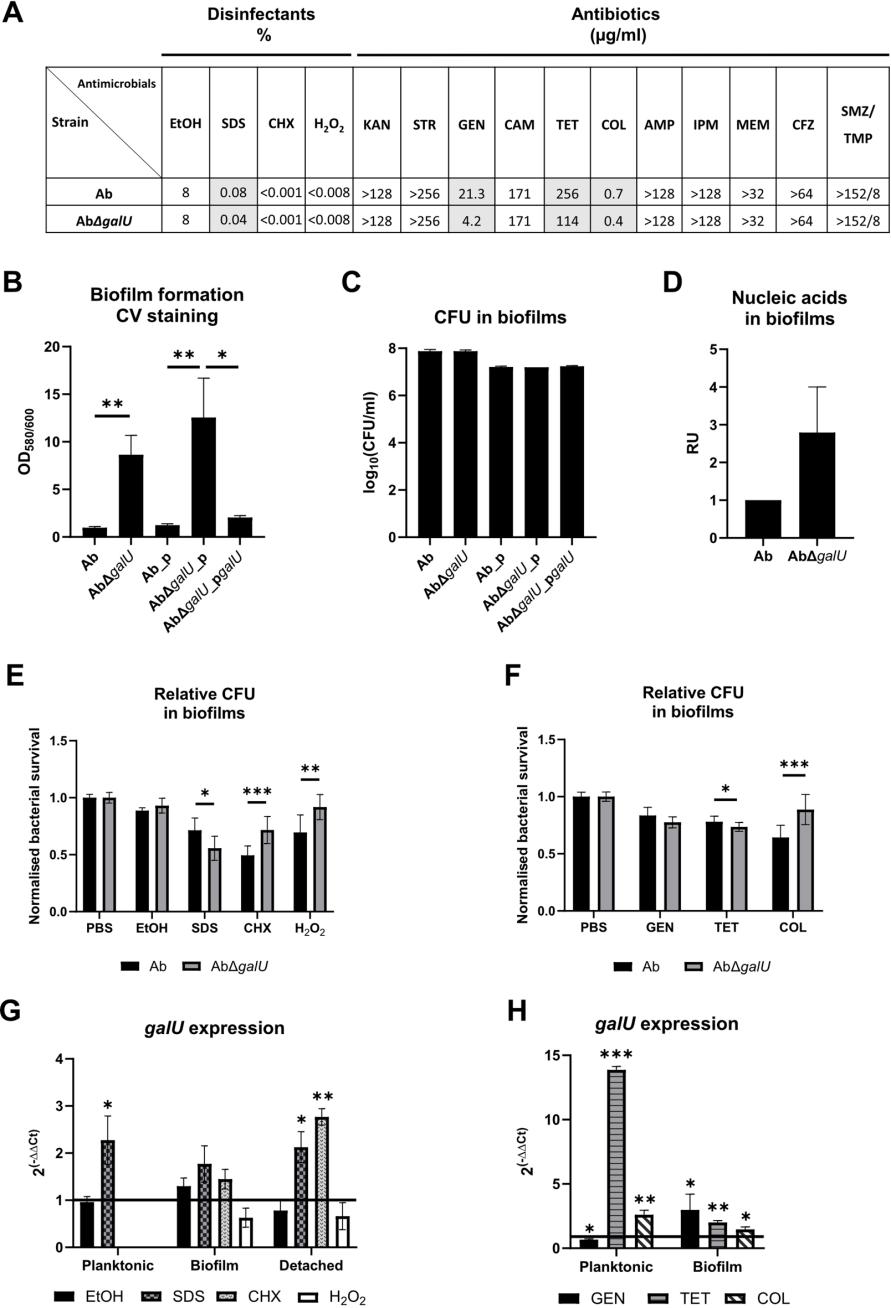
*A. baumannii* resistance to antimicrobials. A – MIC for antimicrobials; SDS – sodium dodecyl sulfate, CHX – chlorhexidine digluconate, KAN – kanamycin, STR – streptomycin, CAM – chloramphenicol, GEN – gentamicin, TET – tetracycline, COL – colistin, AMP – ampicillin, IPM – imipenem, MEM – meropenem, CFZ – cefazolin, SMZ/TMP – sulfamethoxazole and trimethoprim; values in grey demonstrate the changes of MIC comparing mutant and wt strains. B – Biofilm formation was assessed by crystal violet (CV) staining. C – CFU counts in biofilms were counted by serial dilutions. D – Concentrations of nucleic acids were represented as relative units (RU) compared to wt strain. E-F – CFU in biofilms after incubation with antimicrobials were normalised to non-treated controls. 10% EtOH, 0.1% SDS, 0.01% CHX, 1% H_2_O_2_, 6 µg/mL GEN, 170 µg/mL TET, and 15 µg/mL COL were used. G-H – Relative expression of *galU* was assessed by RT-qPCR using *rpoB* gene as a housekeeping gene; Relative expression was normalised to non-treated control; Black line represents non-effected expression level. 1% and 6% EtOH (for planktonic and biofilm/detached), 0.1% SDS, 0.0025% and 0.01% CHX (for biofilm and detached), 0.0625% H_2_O_2_, 14 µg/mL GEN, 140 µg/mL and 170 µg/mL TET (for planktonic and biofilm), 14 µg/mL and 15 µg/mL COL (for planktonic and biofilm) were used. Means with SD are indicated. Significance assessed by t-test, * < 0.05, ** < 0.01, *** < 0.001. Unless otherwise stated, no statistical significance was determined.

In recent years, *A. baumannii* isolates belonging to international clone II (IC II) have become dominant in hospital settings, replacing IC I strains^38^. Thus, we selected a biofilm-forming IC II *A. baumannii* isolate II-a (Ab) and its CPS-deficient mutant (Ab∆*galU*)^39^ for the analysis. The loss of CPS production in mutant strains was confirmed using a Percoll density gradient assay and SDS-PAGE stained with Alcian blue (Fig. S1). We tested whether the CPS-deficient mutant is more sensitive to antimicrobials commonly used for disinfection in hospitals. We also investigated *A. baumannii* susceptibility to different classes of antibiotics. To enable the detection of even minor changes in resistance during the testing of minimal inhibitory concentrations (MIC), antibiotics were diluted in increments smaller than two-fold. Results of MIC testing demonstrated reduced resistance to sodium dodecyl sulfate (SDS), gentamicin, tetracycline, and colistin of the CPS-deficient *A. baumannii* ∆*galU* strain (Fig. 1A). The resistance profiles of ∆*galU* mutant complemented with the *galU* gene cloned into the vector reached a similar level as wild-type (wt) strain (Fig. S2A). Planktonic *A. baumannii* cells were unable to grow even in very low concentrations of chlorhexidine and hydrogen peroxide.

The CPS-deficient mutant exhibited increased surface hydrophobicity compared to the wt, as determined by the salt aggregation test^40^, with cell aggregation observed at ammonium sulphate concentrations of 0.5 M and 1 M, respectively. Next, we investigated how the loss of CPS and increased surface hydrophobicity impact *A. baumannii*’s adhesive properties. Biofilm formation assay showed that the CPS-deficient mutant formed a biofilm approximately 8-fold thicker than the wt, as measured by crystal violet (CV) staining (Fig. 1B). Restoration of the wt phenotype was observed in the complemented strain. CFU enumeration within the biofilms of both wt and mutant strains revealed no significant difference in bacterial load (Fig. 1C). These results suggest that the biofilm of the CPS-deficient mutant contains more extracellular matrix compared to the wt. Indeed, analysis of extracellular matrix extracts revealed approximately 3-fold higher levels of extracellular nucleic acids in the mutant biofilm (Fig. 1D).

Since CPS-deficient mutant forms a thicker biofilm, we decided to investigate if this phenomenon changes the pattern of *A. baumannii* resistance in biofilm. We counted CFU in biofilms after 2 h of exposure to disinfectants and after 24 h of exposure to selected antibiotics. In agreement with the results obtained by MIC determination, the biofilm formed by the CPS-deficient mutant was more sensitive to SDS, demonstrating ~ 22% reduction in CFU counts compared to CFU in biofilms formed by the wt strain (Fig. 1E). Minimal reductions of ~ 6% and ~ 7% were observed for tetracycline and gentamicin, respectively (Fig. 1F). However, *A. baumannii* biofilm lacking CPS was more resistant to chlorhexidine, hydrogen peroxide, and colistin, showing ~ 45%, ~ 32%, and ~ 38% increase of CFU, respectively, probably due to increased thickness of biofilms. No significant difference comparing CFU in biofilms formed by the wt strain and the CPS-deficient mutant was observed after exposure to ethanol. The *galU* complementation showed the tendency of restored CFU in biofilms after the treatments (Fig. S2B).

Next, we analysed the expression of K locus gene *galU* in wt strain during the exposure to the antimicrobials by reverse transcriptase qPCR (RT-qPCR). *galU* gene expression increased ~ 2-fold during the exposure of planktonic cells to SDS (Fig. 1G). While the changes of *galU* expression in biofilms after exposure were not statistically significant, detached bacteria from biofilm demonstrated ~ 2 and ~ 2.5-fold increase of *galU* expression after exposure to SDS and chlorhexidine, respectively. ~14 and ~ 2.5-fold increase in *galU* expression were detected in *A. baumannii* planktonic cells after the treatment with tetracycline and colistin, respectively (Fig. 1H). After the treatment with the same antibiotics, biofilms showed the increase in *galU* expression of ~ 2 and ~ 1.5-fold, respectively. Interestingly, we observed ~ 2-fold reduction of *galU* expression in planktonic cells after the treatment with gentamicin, although ~ 3-fold increase was observed in biofilm. These results demonstrate the protective role of *A. baumannii* CPS during exposure to specific disinfectants and antibiotics, both in planktonic cells and in biofilms.

### CPS protect *A. baumannii* from blue light-induced cytotoxicity and chlorophyllin-induced photodynamic therapy

**Fig. 2:**
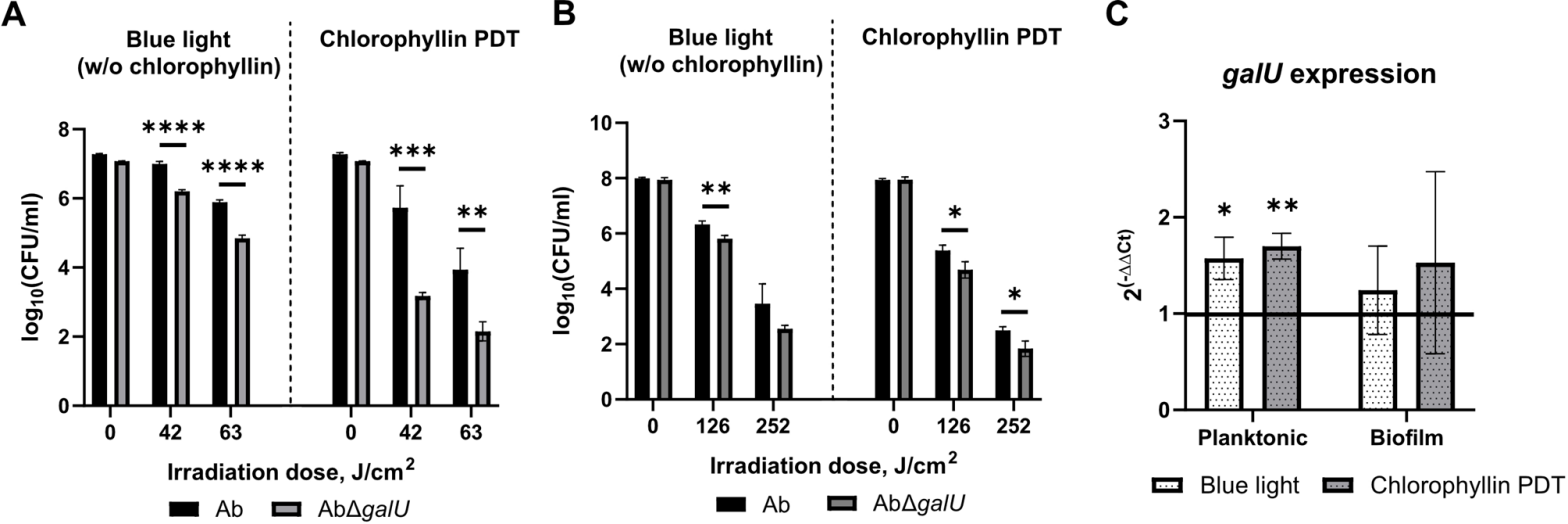
*A. baumannii* resistance to blue light-induced cytotoxicity and chlorophyllin-induced PDT. A – CFU counts of planktonic cells after exposure to blue light or chlorophyllin-induced PDT. B –  CFU counts in biofilms after exposure to blue light or chlorophyllin-induced PDT. C – Relative expression of *galU* was assessed by RT-qPCR using *rpoB* gene as a housekeeping gene; Relative expression was normalised to non-treated control; Black line represents non-effected expression level. 63 J/cm^2^ and 126 J/cm^2^ exposure doses were used for planktonic cells and biofilms, respectively. Means with SD are indicated. Significance assessed by t-test, * < 0.05, ** < 0.01, *** < 0.001, **** < 0.0001.

Next, we investigated whether CPS could play a role in *A. baumannii* resistance to other antibacterial techniques, such as antimicrobial PDT. Chlorophyllin was used as a photosensitizer activated by blue light (402 nm wavelength). Chlorophyllin alone does not show antibacterial activity against *A. baumannii*^41^. Results showed that CPS was an important factor protecting *A. baumannii* from blue light-induced cytotoxicity (samples exposed to blue light only, without chlorophyllin) and chlorophyllin-induced PDT in both planktonic cell cultures (Fig. 2A) and in biofilms (Fig. 2B). Planktonic cells of CPS-deficient mutant were ~ 10-fold more sensitive to blue light-induced cytotoxicity and ~ 100-fold more sensitive to chlorophyllin-induced PDT. As expected, biofilms were more resistant: the biofilm of CPS-deficient mutant was ~ 3-fold more sensitive to blue light-induced cytotoxicity and ~ 4-5-fold more sensitive to chlorophyllin-induced PDT, depending on the irradiation dose. Complemented strain was able to partially restore the resistance to both blue light-induced cytotoxicity and chlorophyllin-induced PDT (Fig. S2C-D).

RT-qPCR experiments demonstrated increased *galU* expression in planktonic cells and in biofilms after both blue light-induced cytotoxicity and chlorophyllin-induced PDT (Fig. 2C). Although gene expression levels increased similarly across two different cellular states (~ 1.5-fold), statistical significance was observed only in planktonic cells due to data variance in biofilm samples.

### CPS provide a protective role during *A. baumannii* exposure to serum and phagocytic cells

**Fig. 3:**
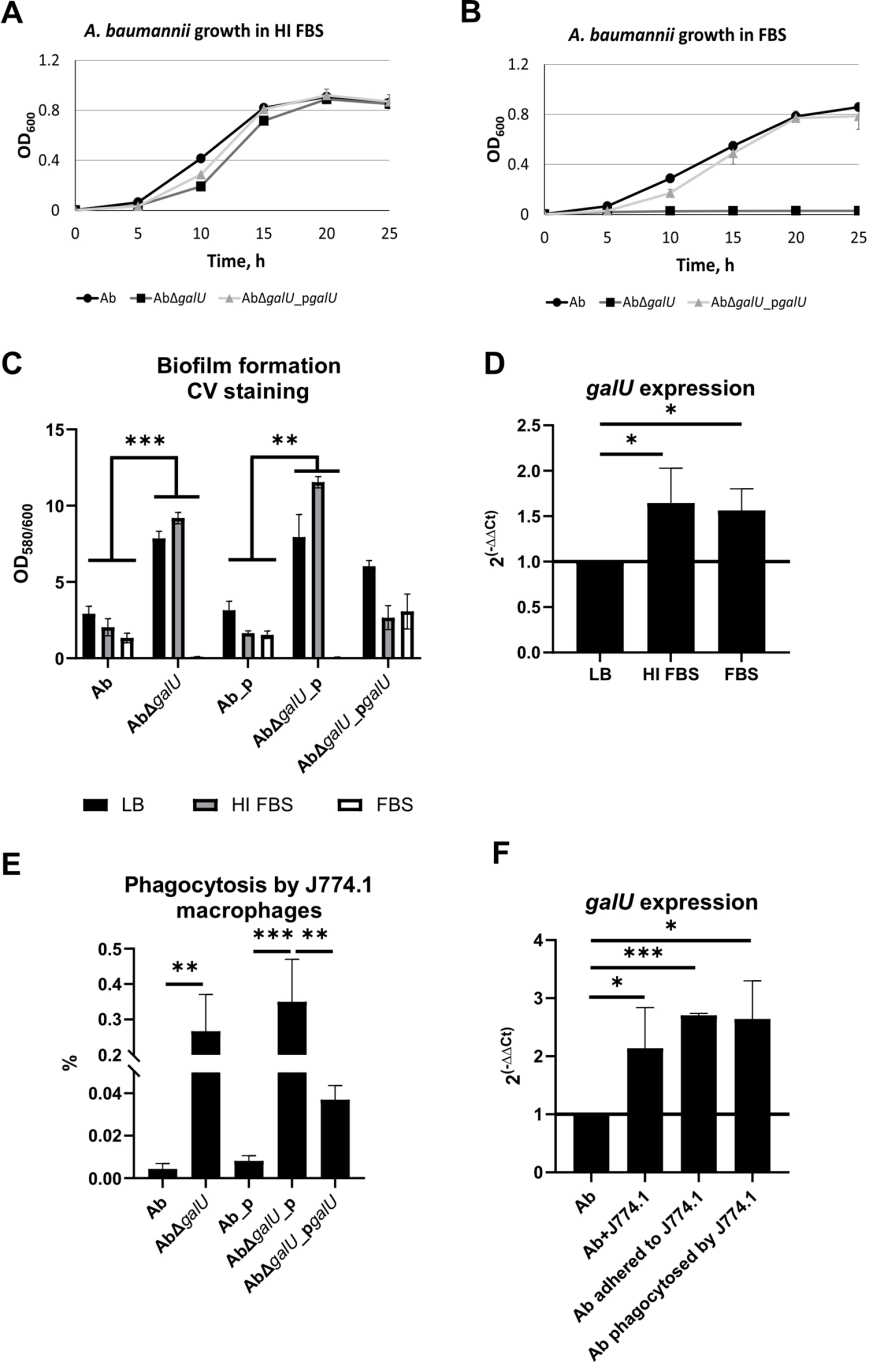
*A. baumannii* resistance to the components of innate immunity. A – *A. baumannii* growth curves in LB media supplemented with 80% heat-inactivated serum. B – *A. baumannii* growth curves in LB media supplemented with 80% serum. C – Biofilm formation in LB media, media supplemented with 50% of heat-inactivated or active serum was assessed by crystal violet (CV) staining. D – Relative expression of *galU* was assessed by RT-qPCR using *rpoB* gene as a housekeeping gene; Relative expression was normalised to non-treated control; Black line represents non-effected expression level. E – Phagocytosis rate by J774.1 macrophages was assessed by gentamicin assay and expressed as % relative to the initial number of bacteria used for infection. F – Relative expression of *galU* was assessed by RT-qPCR using *rpoB* gene as a housekeeping gene; Relative expression was normalised to non-treated control; Black line represents non-effected expression level. Means with SD are indicated. Significance assessed by t-test, *<0.05, **<0.01, ***<0.001.

Investigation of the interaction between *A. baumannii* and innate immune components revealed that the *galU*-deficient strain was unable to grow in active fetal bovine serum (FBS) (Fig. 3A, B). CPS contributed critically to both the growth of planktonic *A. baumannii* cells and to biofilm development under serum-containing conditions (Fig. 3C). RT-qPCR analysis demonstrated increased *galU* expression during *A. baumannii* logarithmic growth in serum (~ 1.5-fold); however, this was not dependent on complement activation, since the comparison of active and heat-inactivated serums showed similar gene expression level (Fig. 3D). *galU*-deficient mutant was ~ 60-fold more sensitive to phagocytosis by J774.1 macrophages (Fig. 3E). Adding J774.1 macrophages into DMEM medium increased *galU* expression in *A. baumannii* by ~ 2-fold compared to *A. baumannii* growing alone (Fig. 3F). An even more pronounced (~ 2.5-fold) increase of *galU* expression was observed when analysing adhered and phagocytosed bacteria (non-phagocytosed bacteria killed with gentamicin). These results show that CPS may play a modulatory role during *A. baumannii* interaction with immune cells.

### CPS-producing *A. baumannii* exhibit reduced proinflammatory response during infection

**Fig. 4:**
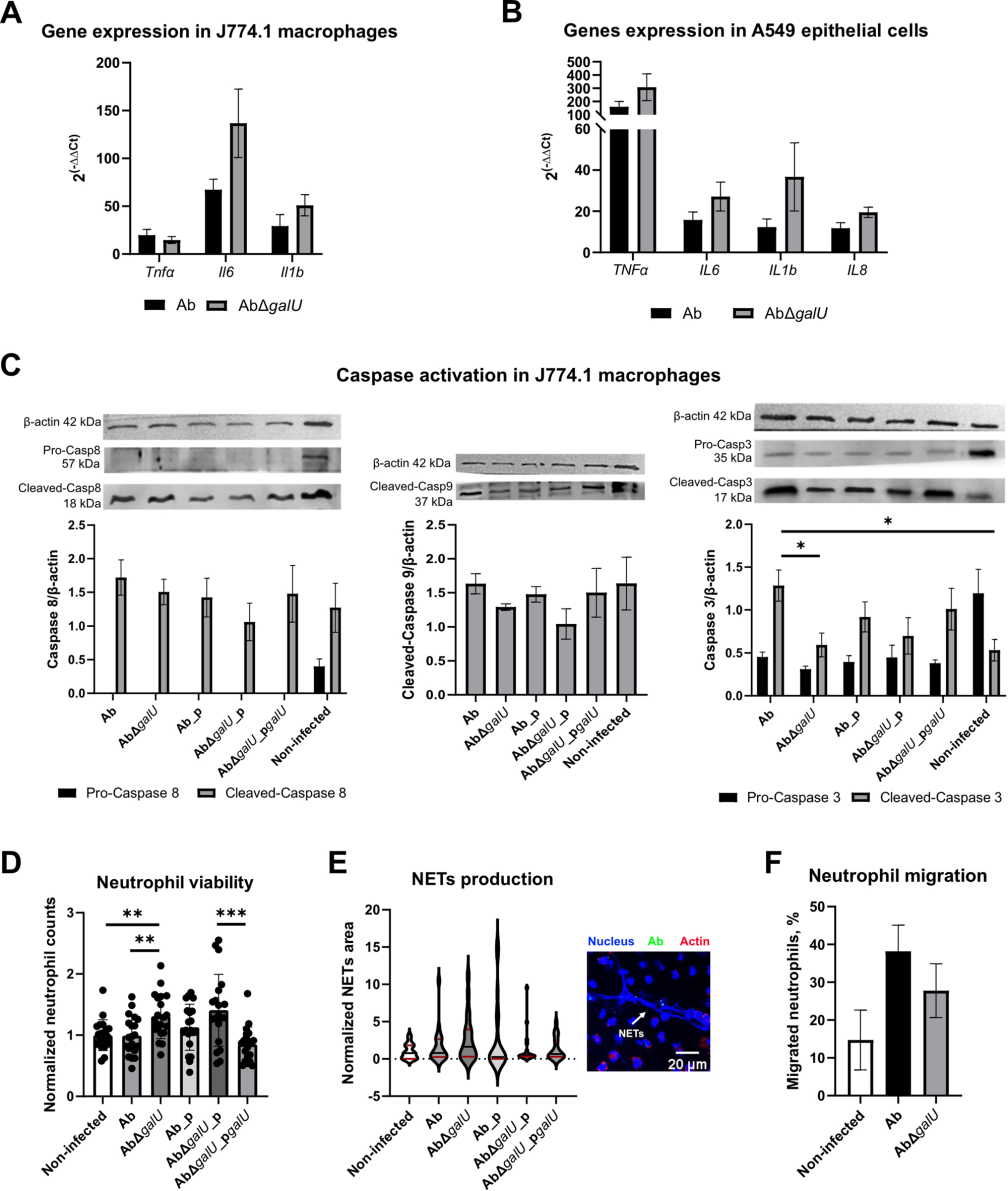
Pro-inflammatory properties of *A. baumannii* strains. A-B – Relative expression after 2 h of incubation was assessed by RT-qPCR using actin as a housekeeping gene; Relative expression was normalised to non-treated control. C – Relative intensity of WB bands was normalised to the expression of actin. D – Counts of human neutrophils were analysed by fluorescent microscopy. E – Violin plot of normalised NETs area (a solid black line indicates median, a solid red line – quartiles); NETs formation was analysed by fluorescent microscopy and expressed as area (µm^2^/cell) of DAPI+ filaments. F – Neutrophil migration was expressed as % relative to the initial number of neutrophils. Means with SEM are indicated. Significance assessed by t-test (C) or Welch’s test (D), * < 0.05, ** < 0.01, *** < 0.001. Unless otherwise stated, no statistical significance was determined.

The proinflammatory effectors TNF-α, IL-6, IL-1β, and IL-8 were investigated for their gene expression in J774.1 macrophages and A549 lung epithelium cells during the infection with CPS-producing Ab and CPS-deficient mutant (Fig. 4A-B). The infection with *A. baumannii* was able to induce a strong inflammatory response in macrophages (Fig. 4A). The expression of *Il6* and *Il1β* showed a tendency to increase in J774.1 macrophages during infection with the CPS-deficient mutant compared to the wt strain. This observation was restored by the complemented strain (Fig. S3A). Analysing gene expression profiles in A549 lung epithelium cells during infection, the tendency of increase for *TNFα*, *IL6*, *IL1β* and *IL8* expression with CPS-deficient mutant compared to wt strain was observed (Fig. 4B). Again, the expression profile was restored by the complemented strain (Fig. S3B).

Next, we analysed the activation of pro-apoptotic caspases in J774.1 macrophages during infection using Western-Blot (WB). In the case of Casp-3 activation, infection with *A.baumannii* induced a switch from inactive form to active form compared to a non-infected control (Fig. 4C). While the decrease of Casp-9 and Casp-8 activation was not significant, CPS-deficient mutant resulted in reduced Casp-3 activation. Consistent results were obtained when human macrophages THP-1 were used for infection (Fig. S3C). This shows that reduction of Casp-3 activation comparing wt and its CPS-deficient mutant can be observed among various macrophage lines.

To analyse how other innate immunity cells, such as neutrophils, interact with Ab and its CPS-deficient mutant, we infected human peripheral blood-purified neutrophils ex vivo. The viability of neutrophils after infection was evaluated by counting the number of cells on fixed samples by fluorescent microscopy. Neutrophil counts were higher in mutant-infected samples compared to wt strain and non-infected sample (Fig. 4D). This indicates that neutrophils are potentially more responsive when exposed to *A. baumannii* strains whose antigenic structures are not shielded by CPS. Therefore, we proceeded to assess the activation properties of neutrophils, such as the production of neutrophil extracellular traps (NETs) and migration. No statistical significance was found in NETs production during *A. baumannii* infection (Fig. 4E). A tendency of increased neutrophil migration through the membrane of Transwells™ inserts upon *A. baumannii* infection was observed (Fig. 4F). Interestingly, migration of neutrophils was slightly reduced in the CPS-deficient *A. baumannii* compared to the wild-type strain.

### CPS have proinflammatory properties and induce neutrophil migration during sterile infection

To analyse how CPS alone modulate immune response, first, we optimised CPS purification from *A. baumannii*. Enzymatic digestion followed by chloroform: phenol extraction (Fig. S4A) and gel filtration (Fig. S4B) were performed. Obtained fractions were analysed for the residues of nucleic acids, lipooligosaccharides (LOS), and lipid A (Fig. S4B-C). Fractions of gel filtration were incubated with J774.1 macrophages, and the cell viability test was performed using MTT method. Fractions 1-6 demonstrated significantly reduced cytotoxicity towards macrophages compared to the sample before gel filtration and fractions 7-8, most likely due to the residues of lipid A in these samples (Fig. S4D). The first two fractions with the purest CPS were used for further analysis.

**Fig. 5:**
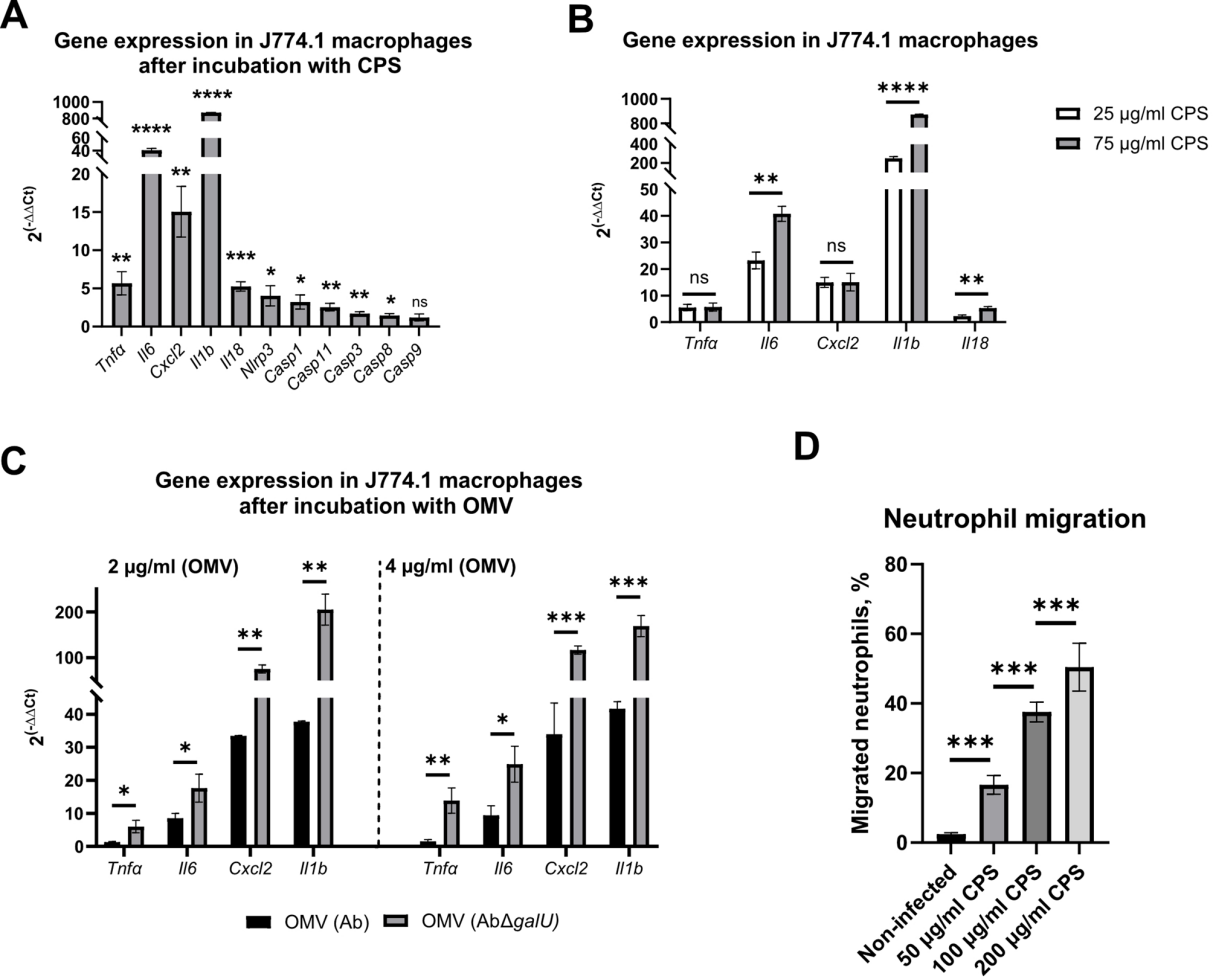
Pro-inflammatory properties of CPS produced by *A. baumannii*. A-C – Relative expression after 24 h of incubation was assessed by RT-qPCR using actin as a housekeeping gene; Relative expression was normalised to non-treated control; 75 µg/mL of purified CPS was used (A). D – Neutrophil migration was expressed as % relative to the initial number of neutrophils. Means with SD are indicated. Significance assessed by t-test, *<0.05, **<0.01, ***<0.001, ****<0.0001.

Purified CPS were incubated with J774.1 macrophages for 24 h. Expression of genes related to apoptosis and pyroptosis, as well as pro-inflammatory cytokines, was analysed by RT-qPCR. All tested genes, except *Casp9*, showed a significant increase in expression, demonstrating the pro-inflammatory capacity of CPS (Fig. 5A). Subsequent analysis of gene expression demonstrated a dose-dependent manner for mRNA expression of IL-1β, IL-6 and IL-18 cytokines; meanwhile, expression of *Cxcl2* and *Tnfα* remained stable, demonstrating independence on the tested CPS concentration (Fig. 5B).

It is known that OMV produced by *A. baumannii* have strong immunomodulatory properties^19^. We decided to test if OMV purified from Ab by ultracentrifugation are covered by CPS. Indeed, SDS-PAGE analysis combined with Alcian blue staining demonstrated that purified OMV fraction contains CPS (Fig. S4E). Next, we investigated whether CPS presence can modulate OMV proinflammatory properties when incubated with J774.1 macrophages. OMV lacking CPS displayed similar cytotoxicity towards macrophages as OMV purified with CPS (Fig. S4F). However, OMV purified from CPS-deficient mutant demonstrated significantly increased proinflammatory properties compared to OMV purified with CPS in all tested conditions using RT-qPCR (Fig. 5C). These results indicate the response-blocking ability of CPS in the presence of OMV.

The expression of MIP2 chemokine by *Cxcl2* gene represented one of the most upregulated responses in J774.1 macrophages stimulated with CPS alone and in combinations with OMV. Thus, we investigated whether CPS has chemoattractant properties for immune cells. For this, we used human peripheral blood neutrophils and Transwells™ inserts. When neutrophils and CPS were placed on the opposite sides of the membrane, the migration of neutrophils towards CPS was observed (Fig. 5D). The migration rates were increased in a dose-dependent manner, confirming the immune-attractant properties of CPS.

## Discussion

Due to multidrug resistance, there is a pressing need to identify novel antibacterial targets against *A. baumannii*. Previously, we demonstrated that the vast majority of clinical *A. baumannii* isolates with different biofilm-forming abilities produce CPS^40^, suggesting that CPS may serve as a promising target for next-generation therapeutics. Nevertheless, the role of CPS in *A. baumannii* pathogenesis – both in the hospital environment and during host infection – remains poorly understood. Previous studies have shown that *A. baumannii* can modulate CPS production via the regulation of K-locus genes through the BfmRS two-component regulatory system^34^. A relationship between increased expression of K-locus genes and CPS production in clinical *A. baumannii* strains has been observed previously^42,43^. In addition, a reversible switch in CPS production has been described, mediated by the insertion of an insertion sequence (ISAba13) into the K locus^44^. These findings indicate that although CPS production is a common trait among *A. baumannii* strains, its expression levels may be conditionally regulated by the bacterium.

Our data demonstrated that the loss of CPS was compensated by the formation of a thicker biofilm, likely due to increased production of extracellular matrix components (e.g. eDNA). This observation is consistent with previous findings in other bacterial species, such as *Klebsiella pneumoniae*, *Mannheimia haemolytica*, and *Pasteurella multocida*, in which CPS-deficient mutants formed thicker biofilms compared to their wt strains^45–47^. It was suggested that CPS interfere with the attachment of bacterial cells to the abiotic surface, resulting in decreased biofilm formation^45^. We observed that *A. baumannii* CPS mutant demonstrated increased surface hydrophobicity, which should hinder bacterial adhesion properties. Indeed, the study on CPS-deficient *A. baumannii* Tn::wzc mutant also demonstrated increased bacterial surface hydrophobicity and increased biofilm biomass as analysed by scanning electron microscopy^48^. Biofilm formation in *A. baumannii* is known to be regulated by the BfmRS two-component system, which also modulates the expression of genes involved in CPS synthesis and Csu pili production^49^. Thus, the enhanced biofilm formation observed in the *galU* mutant may also result from the regulatory effect of BfmRS acting through Csu pili, which are critical for biofilm development. Additionally, pleiotropic effects from the *galU* mutation, which affects UDP-N-acetylglucosamine precursors, could impact poly-N-acetylglucosamine (PNAG) production, a biofilm component that, together with Csu pili, protects *A. baumannii* biofilms from colistin^50^. Interestingly, while CPS loss in ∆*galU* strain either had no effect or increased the susceptibility of planktonic cells to the tested antimicrobial, biofilms formed by the CPS-deficient mutant exhibited increased resistance to certain compounds, including chlorhexidine, hydrogen peroxide, and colistin. These results indicate that the extracellular matrix of biofilm may partially compensate for the absence of CPS. A similar effect was observed for benzalkonium chloride, when the biofilm of the *A. baumannii* CPS-deficient mutant was more resistant than its wt counterpart^48^. Nevertheless, our data show that despite the enhanced matrix production, CPS deficiency negatively affected *A. baumannii* survival under exposure to SDS and tetracycline, in both planktonic and biofilm-associated states. The observed discrepancy could be explained by variations in the mechanisms of action of the antimicrobial agents. Both colistin and chlorhexidine are positively charged molecules. It has been shown that eDNA in *Pseudomonas aeruginosa* biofilms, due to its negative charge, can chelate cations and promote resistance to positively charged antimicrobials such as aminoglycosides and cationic peptides, either directly or via activation of two-component regulatory systems like PhoPQ and PmrAB^51,52^. A similar mechanism may have contributed to the increased resistance of CPS-deficient biofilms of *A. baumannii*, which exhibited elevated levels of nucleic acids. In contrast, the anionic nature of SDS and the amphoteric or slightly negative character of tetracycline were likely not masked by eDNA, allowing these agents to effectively penetrate the biofilm and target bacterial cells.

CPS is increasingly recognised as a promising target for next-generation antimicrobial therapies, including bacteriophages and depolymerases-based approaches, which are often applied in combination with conventional antibiotics^53^. However, our data suggest that caution is warranted, as CPS deficiency – at least in the context of biofilms – may result in increased resistance to certain antimicrobial agents. Other alternative strategies for combating antibiotic-resistant bacteria are also under development, such as antimicrobial PDT. Previous studies have demonstrated that chlorophyllin-mediated PDT can effectively target both planktonic and biofilm-associated *A. baumannii* cells^41,54^. Blue light susceptibility in bacteria, including *A. baumannii*, has been associated with the excitation of endogenous chromophores such as porphyrins and flavins, although the specific mechanisms and identities of these molecules remain incompletely understood^8,55^. In line with these findings, our data show that despite forming thicker biofilms, the CPS-deficient mutant was more sensitive to chlorophyllin-based PDT and blue light exposure compared to wt biofilms. As shown previously, PDT is effective for abiotic surfaces and in chlorophyllin-biopolymer matrices (e.g., chitosan films), which may be further explored for self-sanitising surfaces and coatings for medical devices^56^. Of interest from a biomedical standpoint, chlorophyllin-based PDT may also have a possibility of development for local antimicrobial applications, as blue-light photodynamic systems with a similar mechanism of action have been shown to effectively eliminate *A. baumannii* and other multidrug-resistant bacteria in models of wound infection and hospital-acquired infection^57^.

*A. baumannii* primarily infects immunocompromised hospitalised patients, particularly those undergoing chemotherapy, organ transplantation, those with HIV infection or elderly people^23,24^. In such individuals, components of the adaptive immune system are often the most affected, making the innate immune response especially critical in controlling infection^23^. For this reason, we investigated the role of CPS in *A. baumannii* interactions with human serum and innate immune phagocytic cells such as macrophages and neutrophils. Our findings are consistent with previous studies demonstrating that CPS confers protection to *A. baumannii* against both serum complement and phagocytic killing^40,58,59^. The CPS-deficient mutant strain (Ab∆*galU*) was unable to grow or form biofilms in the presence of active serum. Interestingly, in media supplemented with heat-inactivated serum, the mutant formed thicker biofilms compared to standard LB medium – an effect opposite to that observed for the wt strain. This suggests that under unfavourable conditions, the extracellular matrix of the biofilm may provide a more robust protective function than CPS alone.

In our study, *galU* expression was significantly induced upon the treatment with SDS, chlorhexidine, gentamicin, tetracycline, colistin, and following exposure to blue light, chlorophyllin-induced PDT, FBS, and interaction with macrophages. These findings suggest that *A. baumannii* can dynamically respond to a variety of stresses by upregulating CPS biosynthesis. Previous research has shown that K locus gene expression in *A. baumannii* is regulated by the BfmRS two-component system^34^. Additionally, our data showed that after the treatment with SDS, chlorhexidine, tetracycline, and colistin *galU* expression decreased in biofilm-associated cells compared to planktonic cells. This may be attributed to the protective function of the biofilm extracellular matrix, potentially diminishing the need for enhanced CPS production in sessile communities. The ability of *A. baumannii* to increase *galU* expression in the presence of FBS and during the contact with macrophages indicates that CPS production is a critical factor for the survival of bacteria in the infected host. These findings are in agreement with a previous study showing that human serum albumin altered K-locus gene expression in *A. baumannii*^36^.

Previous studies have indicated that *A. baumannii* strains may activate the host immune response via TLR4-TRIF-IRF3-dependent type I IFN induction^59^. Analysis of pro-inflammatory cytokine expression in J774.1 macrophages and A549 lung epithelial cells revealed that CPS suppresses the inflammatory response during *A. baumannii* infection. A similar effect was reported in a recent study, where IC-I belonging *A. baumannii* CPS-deficient mutant triggered increased IL-6 signalling in a murine infection model^59^. Furthermore, our data demonstrated that the loss of CPS attenuated caspase-3 activation, most likely reducing the extent of non-inflammatory, apoptosis-mediated immune cell death. RT-qPCR data showed higher induction of the pyroptosis marker IL-1β following infection with the *galU* mutant strain compared to the wt, suggesting that bacteria possessing CPS tend to suppress pyroptosis while promoting apoptosis during infection. When evaluating the viability of neutrophils, the proportion of viable cells was comparable between the uninfected control and wt *A. baumannii*-infected samples. In contrast, infection with the CPS-deficient mutant significantly reduced cell death. Collectively, these findings suggest that CPS masks immunogenic bacterial structures, such as outer membrane proteins (OMPs), hindering immune recognition of *A. baumannii*. Capsule removal likely increases OMPs exposure, including OmpA, which is known to induce TNF-α, IL-6, IL-8, and IL-1β expression in A549 cells^60^. Conversely, the absence of CPS exposes antigenic determinants, facilitating immune activation through suppression of apoptosis and enhancement of pro-inflammatory signalling.

Our studies using purified CPS revealed that, although CPS can mask bacterial and OMV-associated antigenic structures during infection, they also possess intrinsic pro-inflammatory properties, as evidenced by their ability to induce pro-inflammatory cytokine gene expression under sterile conditions. This is of particular importance because, as the infection spreads, bacteria may leave molecular traces, such as CPS. Interestingly, our data showed that CPS can induce neutrophil migration. Similarly, serotype K1 CPS of *Porphyromonas gingivalis* induced the migration of naive murine bone marrow-derived polymorphonuclear leukocytes^61^. Moreover, based on our data, neutrophils exhibited more efficient chemotaxis toward capsulated *A. baumannii* compared to the CPS-deficient mutant. These findings suggest that CPS possesses both anti-immunomodulatory and pro-immunomodulatory properties, depending on the stage of infection. While CPS can exhibit pro-inflammatory characteristics, it may simultaneously shield highly immunogenic structures present on intact bacteria or OMV. Similar to OMV, detached CPS may act as immune decoys, diverting immune cell activation away from live bacterial cells.

Multiple studies indicated neutrophil influx as a predominant mechanism of *A. baumannii* clearance during pneumonia^62,63^. Our findings show that *A. baumannii* induces *galU* expression during interaction with J744.1 macrophages, which attenuates the inflammatory response of macrophages compared to the non-capsulated mutant. Nevertheless, CPS promoted neutrophil migration toward *A. baumannii*, while having no significant impact on the level of NETs production – one of the key antimicrobial strategies employed by neutrophils. These results may explain why neutrophils are among the most critical components of innate immunity responsible for the clearance of *A. baumannii*^63^.

A limitation of this study is that only a single *A. baumannii* strain, belonging to IC II, was examined. Including a broader range of strains in future work could clarify whether CPS deficiency produces similar effects across different *A. baumannii* isolates. However, a previous study utilising an IC I strain yielded comparable results in terms of immune cell activation. In addition, our complementary experiments with a *wza* deletion mutant in another *A. baumannii* strain^40^ also indicated that CPS loss increases biofilm resistance to colistin and attenuates caspase-3 activation (Fig. S5). These findings suggest that CPS deficiency may similarly affect multiple *A. baumannii* strains carrying different mutations in CPS production-related genes.

Altogether, this study highlights the critical role of *A. baumannii* CPS in pathogenesis, contributing to both antimicrobial resistance and modulation of innate immune responses. CPS not only masks bacterial antigens but can itself function as a pro-inflammatory signal under sterile conditions. We showed that CPS enhances neutrophil chemotaxis, suggesting CPS as a key modulator of host-pathogen interactions. Targeting CPS biosynthesis represents a promising therapeutic approach to combat antibiotic-resistant *A. baumannii* infections, as our findings show that the innate immune system clears *A. baumannii* infections more effectively in the absence of CPS. Nevertheless, our data indicate that CPS loss may promote biofilm-mediated resistance to certain agents and alter immune signalling, suggesting that future therapeutic strategies should carefully consider drug combinations to maximise treatment efficacy.

## Methods

### Quantification of *A. baumannii* biofilm formation

IC II *A. baumannii* isolate II-a (Ab) and its CPS-deficient mutant (Ab∆*galU*)^39^ were used for the analysis. *A. baumannii* isolate II-a was isolated from a tertiary care hospital in 2010. The biofilm-forming capacity of *A. baumannii* was assessed using a crystal violet staining. An overnight bacterial culture was prepared by inoculating a single colony into 500 µL of LB (Luria-Bertani) broth and incubating at 37 °C. Biofilms were cultivated in 96-well round-bottom plates by adding 200 µL of fresh LB broth and 1 µL of the overnight culture into each well. The plates were incubated for ~ 24 h at 37 °C to allow biofilm development. Subsequently, biofilms were washed with 200 µL PBS. Each well was then stained with 200 µL of 0.1% crystal violet solution for 5 min, followed by three washes with PBS buffer. Biofilm-bound crystal violet was solubilised with 200 µL of 99% ethanol for 5 min. The ethanol was transferred to a flat-bottom 96-well plate for absorbance measurement. Absorbance was measured using a microplate reader (Tecan Infinite M200 Pro) at 600 nm (bacterial growth control) and 580 nm (biofilm quantification). The relative biofilm biomass was expressed as the OD₅₈₀/OD₆₀₀ ratio to normalise for differences in bacterial growth. Bacterial growth was evaluated in heat-inactivated FBS (HI FBS) and active FBS as described previously^40^.

To assess the quantity of extracellular matrix (ECM) components in biofilms, biofilms were mechanically scraped and resuspended in PBS. The suspension was centrifuged at 7,000 × g for 5 min and filtered through a 0.2 μm membrane to remove bacterial cells. ECM components were precipitated by incubating the filtrate with 70% ethanol overnight at − 20 °C. After centrifugation at 12,000 × g for 30 min at 4 °C, the resulting pellet was resuspended in PBS. Nucleic acid concentration was determined using a NanoDrop spectrophotometer.

### Analysis of CPS production

Overnight cultures were centrifuged for 5 min at 7,000 × g and the bacterial pellets were resuspended in 200 µL of PBS. In fresh 1.5 mL microcentrifuge tubes, 900 µL of 50% Percoll (Cytiva, 17089102) density gradient was added, followed by gentle layering of the resuspended cells on top. The tubes were centrifuged at 3,000 × g for 30 min. Capsule production was qualitatively evaluated based on the distribution of cells within the density gradient, as described previously^64^. CPS were visualised by the staining of SDS-PAGE gel with Alcian blue as described previously^40^.

### Characterisation of *A. baumannii* resistance to antimicrobials

The determination of Minimum Inhibitory Concentration (MIC) was performed by diluting the overnight cultures in 2×LB medium to an initial optical density (OD_600_) of 0.01. For MIC determination, 50 µL of each antimicrobial was dispensed into the wells of a 96-well U-bottom microtiter plate. 50 µL of bacterial culture was added to each well. The plate was sealed with parafilm to prevent evaporation and incubated under static conditions at 37 °C for 24 h. Bacterial growth was assessed visually to determine the lowest concentration of antimicrobial that inhibited visible growth, corresponding to the MIC. The concentrations of antimicrobials applied in the following experiments were selected according to the MIC values determined.

For the quantitative assessment of antimicrobial effect, biofilms were incubated with 200 µL of antimicrobials. The plate was incubated statically at 37 °C for 2 h with disinfectants and 24 h with antibiotics. After the treatment, biofilms were washed with PBS and carefully scraped from the well walls and resuspended in PBS. Serial dilutions of the recovered biofilm suspensions were prepared, and colony-forming units (CFU) were enumerated.

### Photodynamic inactivation of *A. baumannii*

PDT on planktonic cells was performed by diluting the overnight culture 1:1,000 in LB. The cultures were grown to OD_600_ of ~ 0.6. 1 mL of culture was centrifuged at 10,000 × g for 10 min, followed by three washes with PBS. Bacterial pellets were resuspended in the same volume of PBS. All subsequent procedures were performed in the dark to prevent premature light activation of chlorophyllin sodium salt (ROTH, 6217.1). Treated samples contained 8.9 mL PBS, 1 mL bacterial suspension, and 0.1 mL of 1.5 mM chlorophyllin solution (final concentration 0.015 mM). Sterile breakable microplate strips were used to aliquot 200 µL for irradiation. Microplate wells were arranged in the center of the irradiation field to ensure even illumination. Samples were irradiated with 402 nm blue light at an intensity of 350 W/m^2^, as described previously^41^. Two irradiation doses were applied: 42 J/cm^2^ and 63 J/cm^2^. Post-irradiation, the antibacterial effect of chlorophyllin-based photodynamic therapy on planktonic *A. baumannii* cells was evaluated via CFU counts.

To evaluate the effect of PDT on biofilms, biofilms were washed three times with PBS. The wells with biofilms were filled with 200 µL of 0.15 mM chlorophyllin solution. Two irradiation doses were applied: 126 J/cm^2^ and 252 J/cm^2^. Biofilms were mechanically disrupted by scraping, and the resulting suspensions were thoroughly mixed for CFU counting. Control biofilms without chlorophyllin were also irradiated under the same conditions. The chlorophyllin concentrations and the irradiation doses are within the range of previously optimised antimicrobial PDT conditions that are effective against Gram-negative bacteria^41,54^. These levels are within the experimentally demonstrated safety range of chlorophyllin-based treatments^65^.

### Purification of OMV

OMV were extracted as described before^19^. Briefly, bacterial cultures were grown in LB medium for 18-20 h at 37 °C, centrifuged at 10,000 × g for 15 min at 4 °C, and supernatants were filtered through a 0.22 μm filter. OMV were collected using ultracentrifugation at 130,000 × g for 3 h at 4 °C. OMV concentration was evaluated using the Bradford assay. Samples were plated on LB plates for sterility control.

### Purification of CPS

CPS were purified as described before^66^ with some modifications. Overnight cultures of *A. baumannii* were centrifuged at 7,000 × g for 5 min. The bacterial pellet was resuspended in 1 mL of lysis buffer (0.29 g TRIS, 1 M 37.8 µL CaCl_2_, 1 M 17.8 mL MgCl_2_, pH 8) and was incubated at 37 °C for 1 h, followed by freezing at − 80 °C for 20 min. 20 µL of DNase I and RNase A were added, and samples were incubated at 37 °C for 30 min. Subsequently, 20 µL of 10% SDS was added to each tube, followed by incubation at 37 °C for 30 min. The samples were then heated at 98 °C for 10 min, and further incubated with 20 µL of lysis buffer containing Proteinase K at 55 °C for 1 h. Samples were centrifuged at 12,000 × g for 2 min to remove precipitants. The phenol extraction was performed by incubating at 70 °C for 15 min, followed by centrifugation at 7,000 × g for 15 min. The aqueous phase was collected and mixed with an equal volume of 96% ethanol and incubated overnight at − 20 °C. Samples were centrifuged at 12,000 × g for 30 min at 4 °C. Ethanol was carefully decanted, and pellets were air-dried for 30 min before resuspending in PBS. Sephadex G-200 gel filtration column (26 cm column height, 40-120 μm bead size) was used to separate CPS from degraded nucleic acids, LOS, and other contaminants. Samples were concentrated with a vacuum concentrator and plated on LB plates for sterility control. Samples were fractionated in 12% SDS-PAGE stained with Alcian blue. The concentration of CPS was evaluated by the Anthrone reaction.

### Infections in vitro

J774.1 and A549 cells (ATCC, CCL-185) were cultivated in DMEM (Gibco, 31966), supplemented with 10% heat-inactivated FBS (Gibco, A5256701) and 1% penicillin-streptomycin (Gibco, 10378). Cells were grown at 37 °C in a humidified atmosphere with 5% CO_2_. Before the infection, cell medium was changed to medium without antibiotics. Subconfluent A549 and J774.1 cells were incubated with bacteria at 37 °C for 2 h without CO_2_ and 24 h with OMV or CPS in the presence of 5% CO_2_. For RT-qPCR analysis, a multiplicity of infection (MOI) of 1:100 (100 bacteria per 1 host cell) was used. Cells were washed with PBS, detached from the surface using a scraper, and centrifuged at 500 × g for 10 min at room temperature. Samples were stored at − 80 °C until RNA extraction.

For WB analysis, subconfluent J774.1 macrophages were infected for 2 h at 37 °C with bacterial suspensions prepared in serum-free DMEM, using MOI of 1:50. THP-1 cells were differentiated in RPMI 1640 medium (Gibco, A10491) with 150 nM phorbol 12-myristate 13-acetate (PMA, Sigma-Aldrich, P1585) for 48 h. Following infection, cells were detached using a scraper and collected by centrifugation at 400 × g for 5 min. Samples were mixed with 4×Laemmli sample buffer and stored at − 80 °C.

Neutrophils were isolated from human peripheral blood, in accordance with the approval of Vilnius Regional Biomedical Research Ethics Committee, no. 2023/11-1551-1011. The experiments were performed in accordance with relevant guidelines and regulations. All volunteers provided written informed consent in accordance with the principles of the Declaration of Helsinki. Neutrophil purification was performed as described previously^67^. Briefly, blood samples were fractionated by centrifugation, the upper plasma layer was separated and the remaining whole blood was treated with dextran to induce erythrocyte sedimentation. The upper leukocyte-containing layer was collected and centrifuged, followed by red blood cell lysis using sequential hypotonic and hypertonic NaCl solutions. After centrifugation, the cell pellet was resuspended in plasma and layered onto a Percoll (Cytiva, 17089102) density gradient, followed by centrifugation. Neutrophils were collected from the bottom of Percoll layer. Neutrophils were suspended in RPMI 1640 at a density of 10^6^ cells/mL and infected with *A. baumannii* at MOI of 1:100 for 1 h. For microscopy, neutrophils were fixed with 4% PFA for 30 min, following the wash with PBS. Neutrophil viability was analysed by counting attached neutrophils to coverslips and compared to the mean of non-infected control.

For neutrophil migration assay, Transwells™ (Costar®, 3464) with 8 μm pores were used. Purified neutrophils (10^6^ cells) were placed on the top of membranes, and *A. baumannii* (or purified CPS) were located on the other side of the membrane at MOI of 1:200. After 2 h of incubation, migrated neutrophils were counted with a hemocytometer.

The viability of eukaryotic cells was determined using the MTT assay, as described before^19^. Cell viability was expressed as a percentage of surviving cells compared to the control sample (without bacteria, OMV or CPS).

Phagocytosis rates by J774.1 macrophages were evaluated using gentamicin assay, as described before^40^. Macrophages were allowed to phagocytose bacteria for 1 h at 37 °C, and phagocytic efficiency was quantified by comparing the CFU recovered from gentamicin-treated (400 µg/ml) macrophages with the initial bacterial count used for infection.

### Evaluation of gene expression by RT-qPCR

Planktonic bacterial samples were grown with disinfectants for 24 h at 37 °C. Planktonic bacteria (OD_600_ 0.6) were treated with antibiotics for 4 h at 37 °C. Biofilms were incubated with antimicrobials for 24 h at 37 °C. After incubation, detached bacteria from biofilms were collected by centrifuging supernatants, and biofilms were scraped carefully from the plastic. For serum stress, bacteria were grown in 50% of heat-inactivated or active FBS until the middle of the exponential phase (OD_600_ 0.4-0.7). Bacteria were centrifuged at 5,000 × g for 5 min. For macrophage stress, 9.6 × 10^6^ of J774.1 macrophages were infected with bacteria using MOI 1:100 and incubated in serum-free DMEM for 14 h (without CO_2_). Supernatants with bacteria were collected (Ab+J774.1), and cells (Ab adhered to J774.1) were washed with DPBS. For the evaluation of phagocytosed bacteria (Ab phagocytosed by J744.1), cells were additionally treated with 400 µg/mL gentamicin for 30 min. Cells were detached from the surface using a scraper and centrifuged at 400 × g for 10 min. All samples were stored at − 80 °C until RNA extraction.

RNA was isolated using the GeneJET RNA Purification Kit (Thermo Fisher Scientific), and cDNA synthesis was performed using the Maxima H Minus First Strand cDNA Synthesis Kit with dsDNase (Thermo Fisher Scientific). Oligo(dT) primers and random hexamer primers were used for eukaryotic and bacterial gene expression analysis, respectively. Gene expression was analysed by quantitative PCR (RT-qPCR) using DreamTaq polymerase and SYTO-9 fluorescent dye (Thermo Fisher Scientific) with amplification carried out on the CFX Real-Time System (Bio-Rad), or Maxima SYBR Green RT-qPCR Master Mix (Thermo Fisher Scientific) using Applied Biosystems QuantStudio3 Real-time PCR system. The primers used for RT-qPCR are detailed in Table S1. All procedures for RNA purification, cDNA synthesis, and RT-qPCR were conducted following the manufacturer’s recommendations. Relative gene expression was calculated using the ∆∆Ct method, with *rpoB* or actin used as the reference genes.

### Western-Blot analysis

6 µg of total proteins from cell lysates were loaded on 10% SDS-PAGE gel. Proteins were then transferred onto a 0.2 μm nitrocellulose membrane (Whatman^®^, 10401396) using semi-dry transfer. Membranes were blocked with 5% non-fat dry milk in TBS-T buffer for 1 h, followed the washes with TBS-T. Membranes were incubated with primary antibodies overnight at 4 °C. After washing in TBS-T, membranes were incubated with appropriate secondary antibodies for 1 h at room temperature. Final washes were performed, and the signals were visualised using the UVITEC Alliance Q9 Advanced imaging system (UVITEC, UK) or Bio-Rad Molecular Imager Gel Doc XR+ transilluminator (Bio-Rad, USA). β-actin was detected using the chromogenic reagent 1-Step™ TMB-Blotting Substrate Solution (Thermo Fisher Scientific, USA), while other targets were visualised using the chemiluminescent SuperSignal™ West Atto Ultimate Sensitivity Substrate (Thermo Fisher Scientific, USA). Band intensities were analysed using ImageJ software, and target protein expression levels were normalised to β-actin. The following primary antibodies were used at the indicated dilutions: GTX33610 (GeneTex) against β-actin (42 kDa, 1:5,000); #9746 against pro-Caspase-8 (57 kDa) and cleaved Caspase-8 (18 kDa, 1:500); #9502 against cleaved Caspase-9 (37 kDa, 1:1,000); #9661 against cleaved Caspase-3 (17 kDa, 1:1,500); #9662 against pro-Caspase-3 (35 kDa, 1:2,000) (Cell Signaling Technology). Secondary antibodies: anti-rabbit IgG, HRP-linked (#7074S) and anti-mouse IgG, HRP-linked (#7076) (Cell Signaling Technology, 1:5,000).

### Immunofluorescent microscopy

For microscopy, GFP-producing *A. baumannii* were used^68^. Fixed samples were permeabilised with 0.1% Triton X-100 and washed with PBS. Samples were incubated with DAPI (Sigma-Aldrich, D9542; 1:1,000) and Phalloidin-iFluor 647 (Abcam, 176759; 1:1,000) for 30 min. Coverslips were washed with PBS and mounted with ProLongGold (ThermoFisher Scientific). Cells were visualised under the confocal microscope (Leica SP8) with 63x oil-immersed objective. NETs were identified as DAPI-stained extracellular fibers. The area occupied by NETs and the number of viable neutrophils were quantitatively assessed. Viable neutrophils per picture were counted and normalised to the non-infected control. NETs area (µm^2^/cell) per picture was normalised to the non-infected control. Microscopy images were analysed with ImageJ software.

### Statistical analysis

Data were analysed using Prism 8 software (GraphPad). Student’s t-test or Welch’s test was performed to analyse the datasets from at least 3 biological replicates with 3 technical replicates unless otherwise stated.

## Supplementary Information

Below is the link to the electronic supplementary material.

Supplementary Material 1

## Data Availability

Data are available upon request by contacting Jurate Skerniskyte ( jurate.skerniskyte@gmc.vu.lt ).
