# Supplementary material for: Capsular polysaccharides of Acinetobacter baumannii modulate antimicrobial resistance and innate immune response
Source: Sci Rep. 2026 Mar 21;16:14478. doi: 10.1038/s41598-026-44001-w (PMC13150009; doi:10.1038/s41598-026-44001-w)
Supplement: Supplementary file 1 — Supplementary Material 1 [file 41598_2026_44001_MOESM1_ESM.docx]

**Supporting Information**

**Capsular Polysaccharides of *Acinetobacter baumannii* Modulate Antimicrobial Resistance and Innate Immune Response**

**Laurita Klimkaite^1^, Giedre Kukanauskaite^1^, Joris Naujalis^1^, Danas Ivanauskas^1^, Dominykas Grigorjevas^1^, Greta Petrenaite^1^, Meda Skinkyte^1^, Jekaterina Porchun^1^, Egle Zalyte^1^, Irina Buchovec^2^, Jurate Skerniskyte^1*^**

**Affiliation**

^1^ Institute of Biosciences, Life Sciences Center, Vilnius University, Vilnius, 10257, Lithuania

^2^ Institute of Photonics and Nanotechnology, Faculty of Physics, Vilnius University, Vilnius, 10222, Lithuania

*** Correspondence:**

**Jurate Skerniskyte**

**E-mail: jurate.skerniskyte@gmc.vu.lt**

**Supplementary Table S1. Oligonucleotides used in this study.**

| **Primer name** | **Primer sequence 5' – 3'** | **Primer function** | **Reference** |
| --- | --- | --- | --- |
| MuacF | TGTCCACCTTCCAGCAGATGT | Evaluation of mouse β-actin coding gene expression | (Skerniškytė et al., 2021) |
| MuacR | TCAGTAACAGTCCGCCT |  |  |
| MutnF | AGGAGAAAGTCAACCTCCT | Evaluation of mouse *Tnf* gene expression | (Skerniškytė et al., 2021) |
| MutnR | AAAGTAGACCTGCCCGGAC |  |  |
| Mu6F | TCCAGTTGCCTTCTTGGGAC | Evaluation of mouse *Il6* gene expression | (Skerniškytė et al., 2021) |
| Mu6R | GTACTCCAGAAGACCAGAGG |  |  |
| mNLRP3 RT fwd | CGAGACCTCTGGGAAAAAGCT | Evaluation of mouse *Nlrp3* gene expression | (Skerniškytė et al., 2021) |
| mNLRP3 RT rev | GCATACCATAGAGGAATGTGATGTACA |  |  |
| mCaspase-1 RT fwd | TTTCAGTAGCTCTGCGGTGT | Evaluation of mouse *Casp1* gene expression | (Skerniškytė et al., 2021) |
| mCaspase-1 RT rev | TTTCTTCCTGATTCAGCACTCTC |  |  |
| mCaspase-11 RT fwd | GCCACTTGCCAGGTCTACGAG | Evaluation of mouse *Casp11* gene expression | (Skerniškytė et al., 2021) |
| mCaspase-11 RT rev | AGGCCTGCACAATGATGACTTT |  |  |
| IL18F | AGGACACTTTCTTGCTTGCC | Evaluation of mouse *Il18* gene expression | (Skerniškytė et al., 2021) |
| IL18R | CACAAACCCTCCCCACCTAA |  |  |
| IL-1betaF | TGGACCTTCCAGGATGAGGACA | Evaluation of mouse *Il1b* gene expression | (Skerniškytė et al., 2021) |
| IL-1betaR | GTTCATCTCGGAGCCTGTAGTG |  |  |
| IL-10R | CGGTTAGCAGTATGTTGTCCAGC | Evaluation of mouse *Il10* gene expression | (Tian et al., 2016) |
| IL-10F | CGGGAAGACAATAACTGCACCC |  |  |
| MIP-2F | CATCCAGAGCTTGAGTGTGACG | Evaluation of mouse *Cxcl2* gene expression | (Saup et al., 2023) |
| MIP-2R | GGCTTCAGGGTCAAGGCAAACT |  |  |
| Casp3F  Casp3R | GGAGTCTGACTGGAAAGCCGAA  CTTCTGGCAAGCCATCTCCTCA | Evaluation of mouse *Casp3* gene expression | (Ding et al., 2021) |
| Casp8F  Casp8R | ATGGCTACGGTGAAGAACTGCG  TAGTTCACGCCAGTCAGGATGC | Evaluation of mouse *Casp8* gene expression | (Feng et al., 2024) |
| Casp9F  Casp9R | AGTTCCCGGGTGCTGTCTAT  GCCAATGGTCTTTCTGCTCAC | Evaluation of mouse *Casp9* gene expression | (Cho et al., 2023) |
| act_hum_F | CACCATTGGCAATGAGCGGTTC | Evaluation of human actin coding gene expression | (Lahlali et al., 2016) |
| act_hum_R | AGGTCTTTGCGGATGTCCACGT |  |  |
| hTNFa_F | CCTGTGAGGAGGACGAACAT | Evaluation of human *TNF* gene expression | (Žitkutė, 2024) |
| hTNFa_R | GGTTGAGGGTGTCTGAAGGA |  |  |
| hIL6_F | AGCCCTGAGAAAGGAGACAT | Evaluation of human *IL6* gene expression | (Žitkutė, 2024) |
| hIL6_R | TTTCAGCCATCTTTGGAAGG |  |  |
| Il-1b_hum_F | CCACAGACCTTCCAGGAGAATG | Evaluation of human *IL1B* gene expression | (Jiang et al., 2023) |
| Il-1b_hum_R | GTGCAGTTCAGTGATCGTACAGG |  |  |
| CXCL8_F | GAAGGTGCAGTTTTGCCAAG | Evaluation of human *CXCL8* gene expression | (Žitkutė, 2024) |
| CXCL8_R | GGTCCACTCTCAATCACTCTCAG |  |  |
| hIL12_F | GACCTCTTTCATAACTAATGGGAGT | Evaluation of human *IL12A* gene expression | (Žitkutė, 2024) |
| hIL12_R | CTAAGGCACAGGGCCATCAT |  |  |
| IL18_F | GATAGCCAGCCTAGAGGTATGG | Evaluation of human *IL18* gene expression | (Jiang et al., 2023) |
| IL18_R | CCTTGATGTTATCAGGAGGATTCA |  |  |
| Casp1_hum_F | GCTGAGGTTGACATCACAGGCA | Evaluation of human *CASP1* gene expression | (Jiang et al., 2023) |
| Casp1_hum_R | TGCTGTCAGAGGTCTTGTGCTC |  |  |
| Casp4_hum_F | GGGATGAAGGAGCTACTTGAGG | Evaluation of human *CASP4* gene expression | (Jiang et al., 2023) |
| Casp4_hum_R | CCAAGAATGTGCTGTCAGAGGAC |  |  |
|  |  |  |  |
| rpoB_qF  rpoB_qR | CGATTCGTACAGAACATTCTT  TAAAGCAGCATTGCCAGAATA | Evaluation of *A. baumannii rpoB* gene expression | (Armalyte et al. 2023) |
| galUF1  galUR1 | AGGCTCTGCACCATCAAACT  ACCTTTCGGCGTGTTCTCAA | Evaluation of *A. baumannii galU* gene expression | This study |

Supplementary references:

Jiang, Q. et al. Inflammasomes in rheumatoid arthritis: A pilot study. *BMC Rheumatology*, *7*(1), 39 (2023). 10.1186/s41927-023-00353-8

Kielkopf, C. L., Bauer, W., & Urbatsch, I. L. Bradford Assay for Determining Protein Concentration. *Cold Spring Harbor Protocols*, *2020*(4), 102269 (2020). 10.1101/pdb.prot102269

Lahlali, T. et al. Netrin-1 Protects Hepatocytes Against Cell Death Through Sustained Translation During the Unfolded Protein Response. *Cellular and Molecular Gastroenterology and Hepatology*, *2*(3), 281-301.e9 (2016). 10.1016/j.jcmgh.2015.12.011

Livak, K. J., & Schmittgen, T. D. Analysis of relative gene expression data using real-time quantitative PCR and the 2(-Delta Delta C(T)) Method. *Methods (San Diego, Calif.)*, *25*(4), 402–408 (2001). 10.1006/meth.2001.1262

Saup, R. et al. Increased Circulating Osteopontin Levels Promote Primary Tumour Growth, but Do Not Induce Metastasis in Melanoma. *Biomedicines*, *11*(4), Article 4 (2023). 10.3390/biomedicines11041038

Skerniškytė, J., Karazijaitė, E., Lučiūnaitė, A., & Sužiedėlienė, E. OmpA Protein-Deficient Acinetobacter baumannii Outer Membrane Vesicles Trigger Reduced Inflammatory Response. *Pathogens*, *10*(4), Article 4 (2021). 10.3390/pathogens10040407

Skerniškytė, J., Krasauskas, R., Péchoux, C., Kulakauskas, S., Armalytė, J., & Sužiedėlienė, E. Surface-Related Features and Virulence Among Acinetobacter baumannii Clinical Isolates Belonging to International Clones I and II. *Frontiers in Microbiology*, *9* (2019). 10.3389/fmicb.2018.03116

Tian, Y. et al. Mesenchymal stem cells improve mouse non-heart-beating liver graft survival by inhibiting Kupffer cell apoptosis via TLR4-ERK1/2-Fas/FasL-caspase3 pathway regulation. *Stem Cell Research & Therapy*, *7*(1), 157 (2016). 10.1186/s13287-016-0416-y

Žitkutė, V. Atsparumo 5-fluoruracilui ir oksaliplatinai molekulinių mechanizmų tyrimai žmogaus kolorektalinės karcinomos ląstelėse [Vilniaus universiteto leidykla] (2024). 10.15388/vu.thesis.593

Ding, G. et al. Silenced lncRNA DDX11-AS1 or up-regulated microRNA-34a-3p inhibits malignant phenotypes of hepatocellular carcinoma cells via suppression of TRAF5. *Cancer Cell Int*. 21(1):179 (2021). 10.1186/s12935-021-02360-6.

Feng, Y. et al. Ferroptosis-related biomarkers for adamantinomatous craniopharyngioma treatment: conclusions from machine learning techniques. *Front Endocrinol (Lausanne)*. 15:1362278 (2024). 10.3389/fendo.2024.1362278.

Cho, E. et al. Tumor-targeted liposomes with platycodin D2 promote apoptosis in colorectal cancer. *Mater Today Bio*. 22:100745 (2023). 10.1016/j.mtbio.2023.100745.

Armalytė, J. et al. A polyamine acetyltransferase regulates the motility and biofilm formation of Acinetobacter baumannii. *Nat Commun*. 14(1):3531 (2023). 10.1038/s41467-023-39316-5.


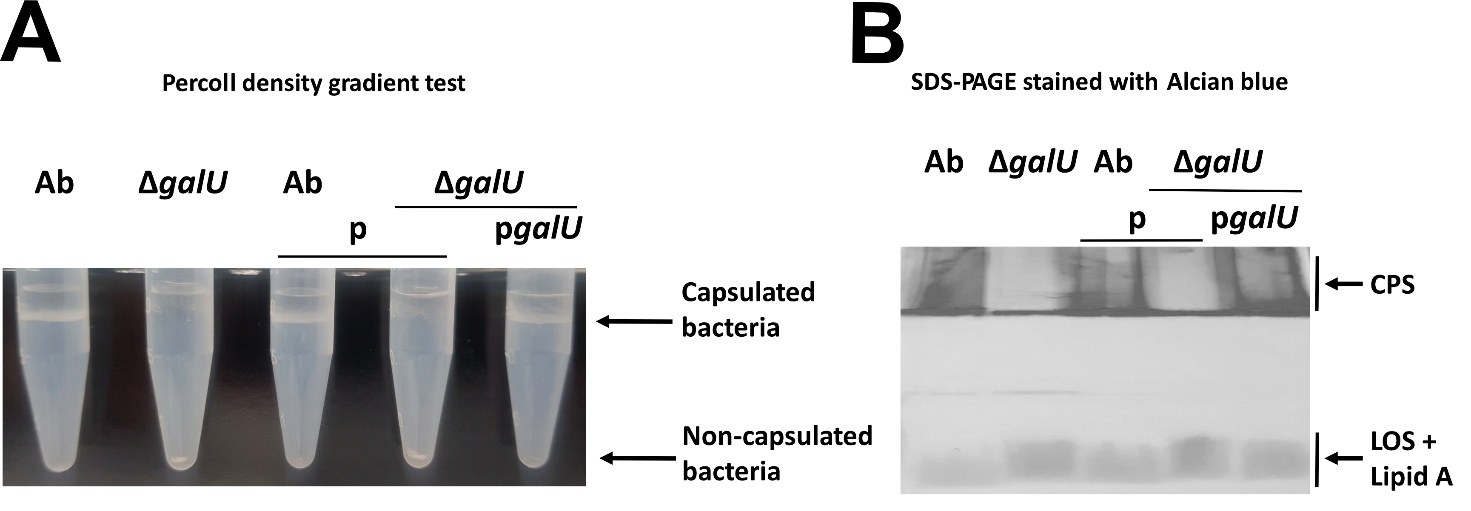


**Supplementary Figure S1.** The loss of CPS production was assessed by Percoll density gradient test (A) and SDS-PAGE analysis with Alcian blue staining (B).


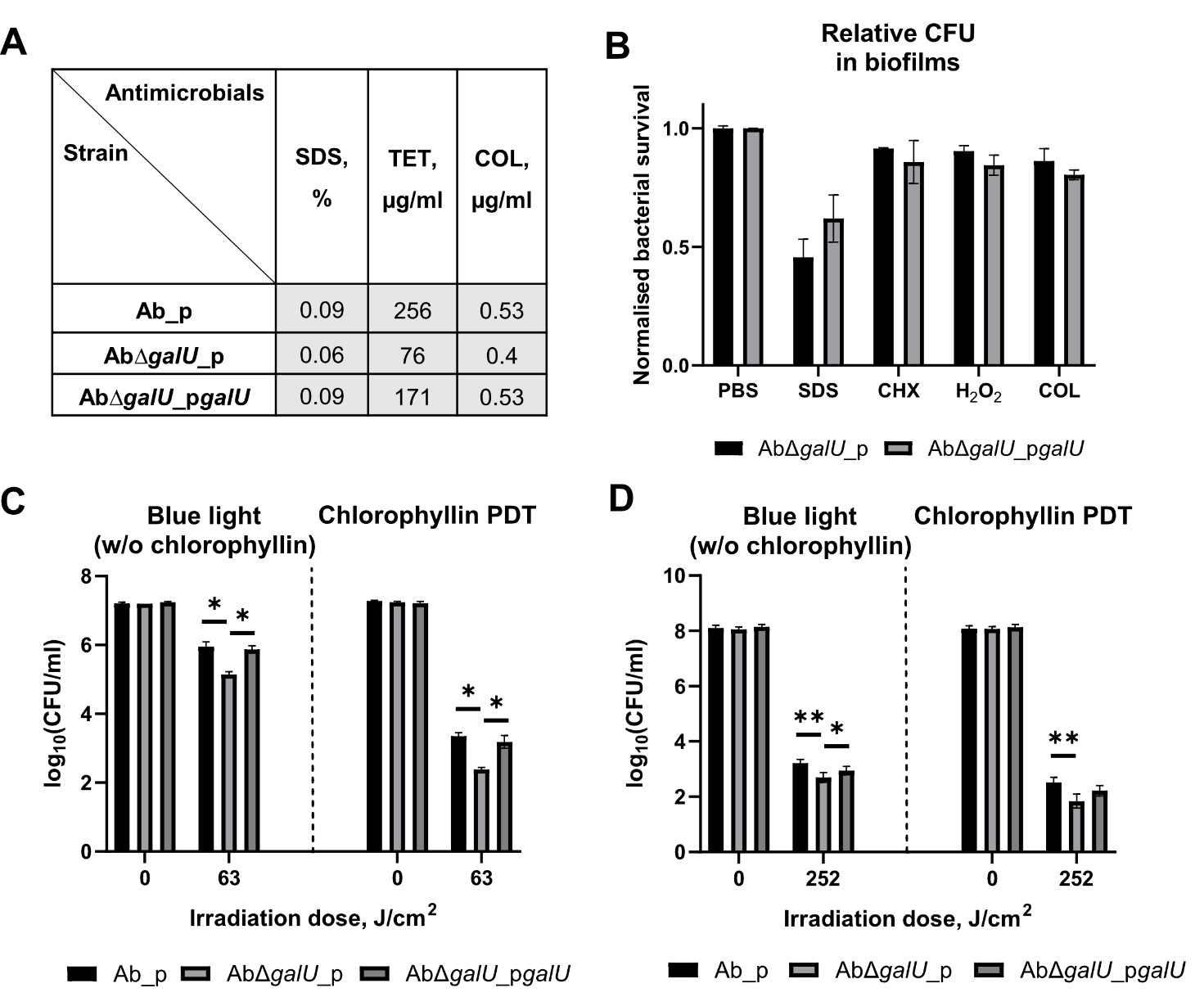


**Supplementary Figure S2.** A-B – The impact of *galU* complementation on MIC (A) and CFU in biofilms (B). 0.05% SDS, 0.01% CHX, 0.5% H_2_O_2_ were used for biofilms assays. C-D – CFU counts of planktonic cells (C) and in biofilms (D) after exposure to blue light or chlorophyllin-induced PDT. Means with SD are indicated from 3 replicates. Significance assessed by t-test, *<0.05, **<0.01. Unless otherwise stated, no statistical significance was determined.


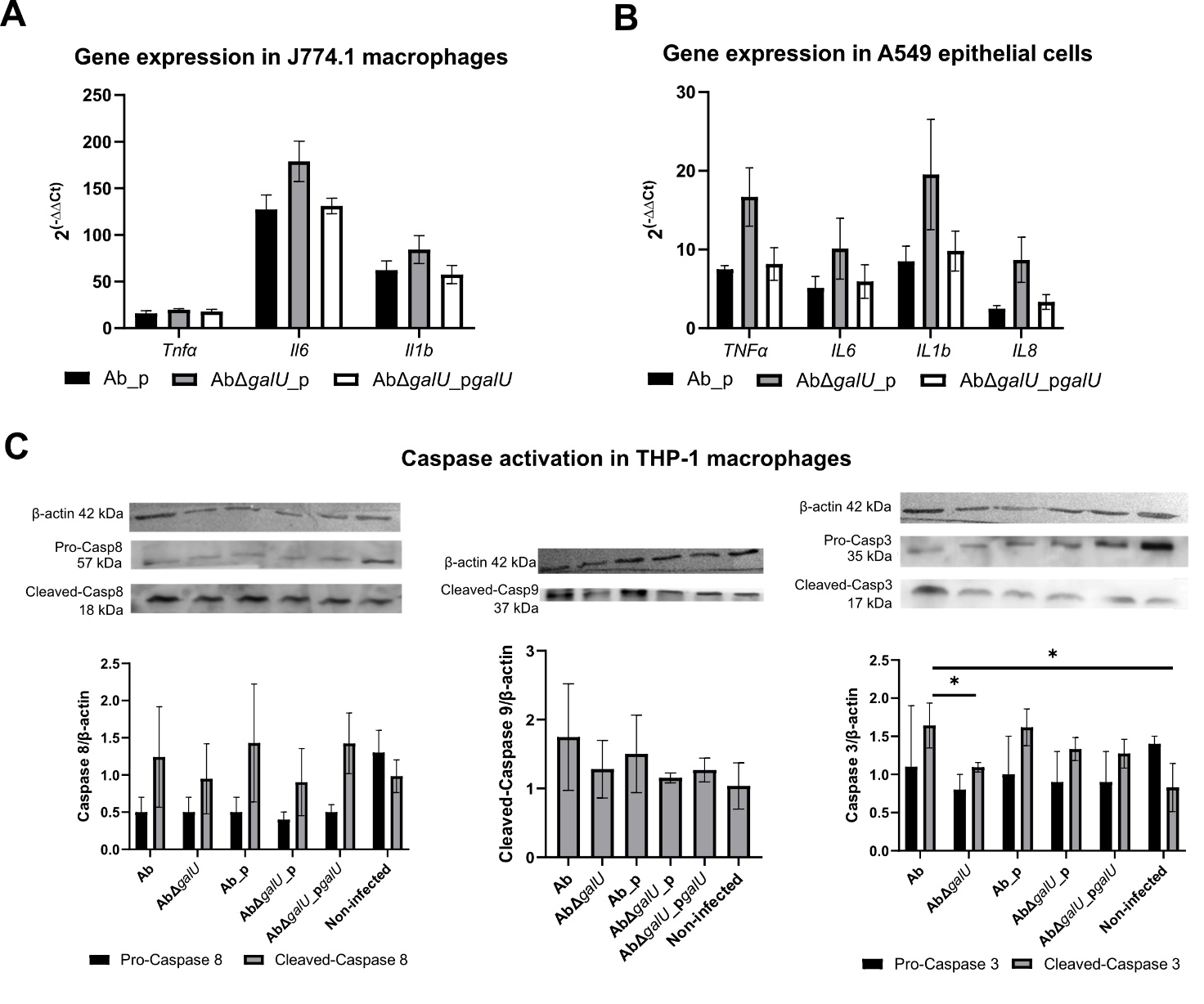


**Supplementary Figure S3.** A-B – Relative expression after 2 h of incubation was assessed by qPCR using actin as a housekeeping gene. Relative expression was normalised to non-treated control. Means with SEM are indicated. C - Relative intensity of WB bands was normalised to the expression of actin. Means with SD are indicated. Significance assessed by t-test, *<0.05. Unless otherwise stated, no statistical significance was determined.


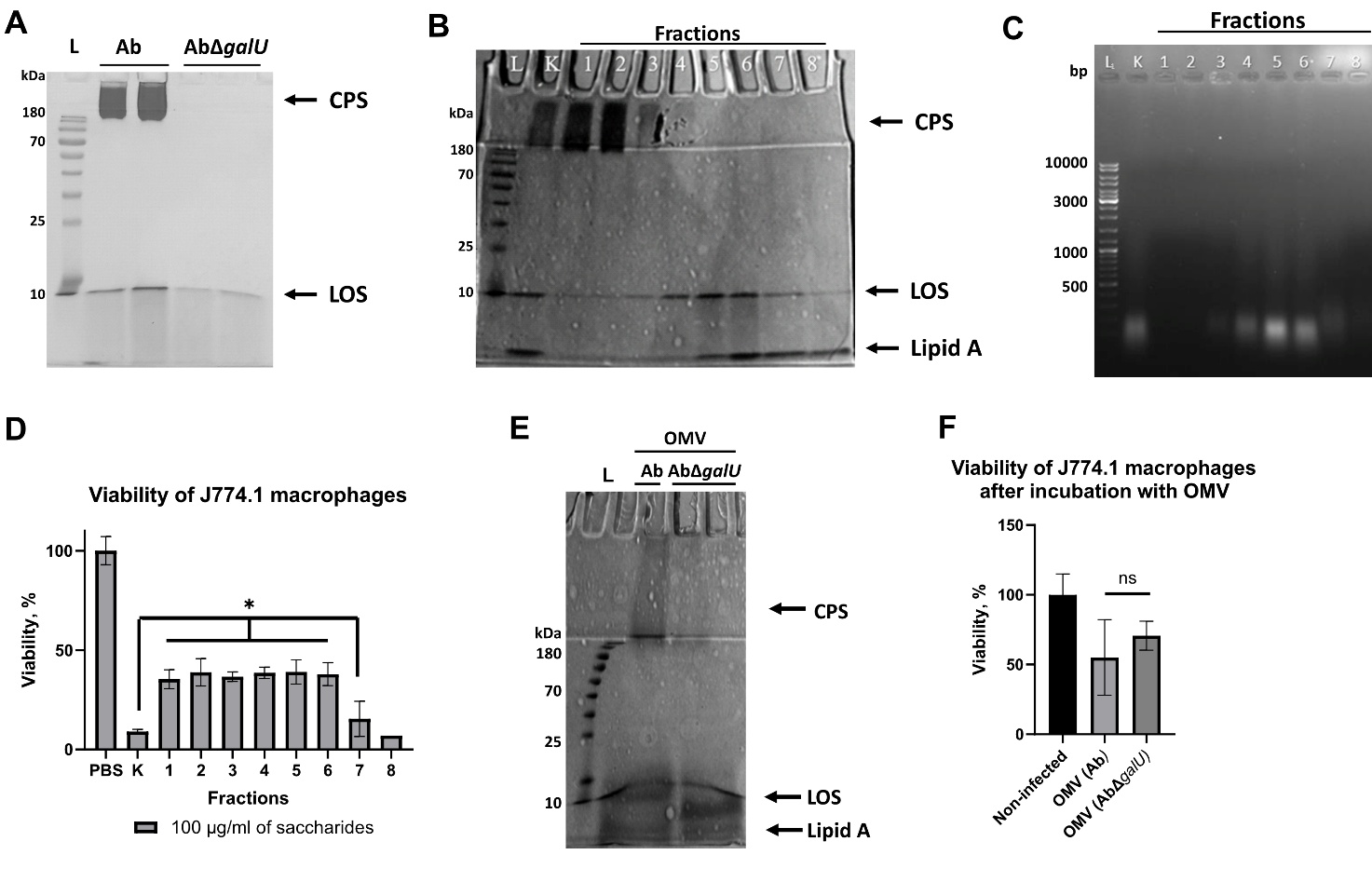


**Supplementary Figure S4.** A – CPS purified from Ab and Ab∆*galU* before gel filtration fractionated in SDS-PAGE and stained with Alcian blue. B – Fractions of Ab collected after gel filtration fractionated in SDS-PAGE and stained with Alcian blue; K-sample before gel filtration. C – Fractions collected after gel filtration fractionated in 1 % agarose gel and stained with ethidium bromide; K-sample before gel filtration. D – Viability of J774.1 macrophages after incubation with saccharides for 24 h was assessed by MTT method. Only one measurement was performed with fraction 8. E – OMV loaded in SDS-PAGE and stained with Alcian blue. F – Viability of J774.1 macrophages after 24 h of incubation was assessed by MTT method. Means with SD are indicated from 3 replicates. Significance assessed by t-test, ns – non-significant, *<0.05. L – PageRuler Prestained Protein Ladder (ThermoFisher Scientific, #26616); L1 – GeneRuler DNA Ladder Mix (ThemoFisher Scientific, #SM0334).


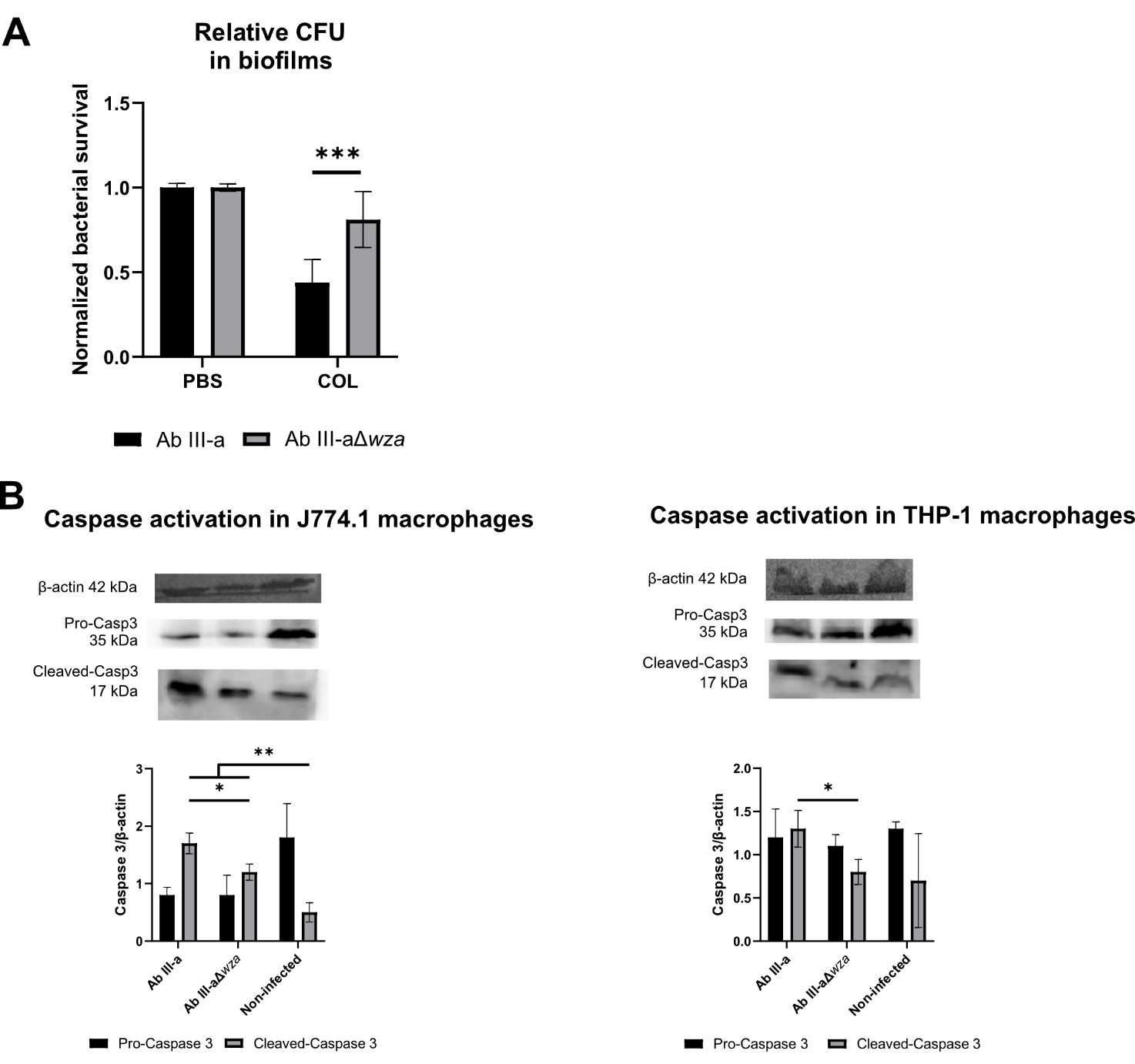


**Supplementary Figure S5.** The effect of *wza* deletion in *A. baumannii* III-a strain on biofilm resistance to colistin (A) and Casp-3 activation in J774.1 and THP-1 macrophages (B). A – CFU in biofilms after incubation with antimicrobials were normalised to non-treated controls. 15 µg/mL colistin was used. B – Relative intensity of WB bands was normalised to the expression of actin. Means with SD are indicated. Significance assessed by t-test, *<0.05, **<0.01, ***<0.001. Unless otherwise stated, no statistical significance was determined.

**
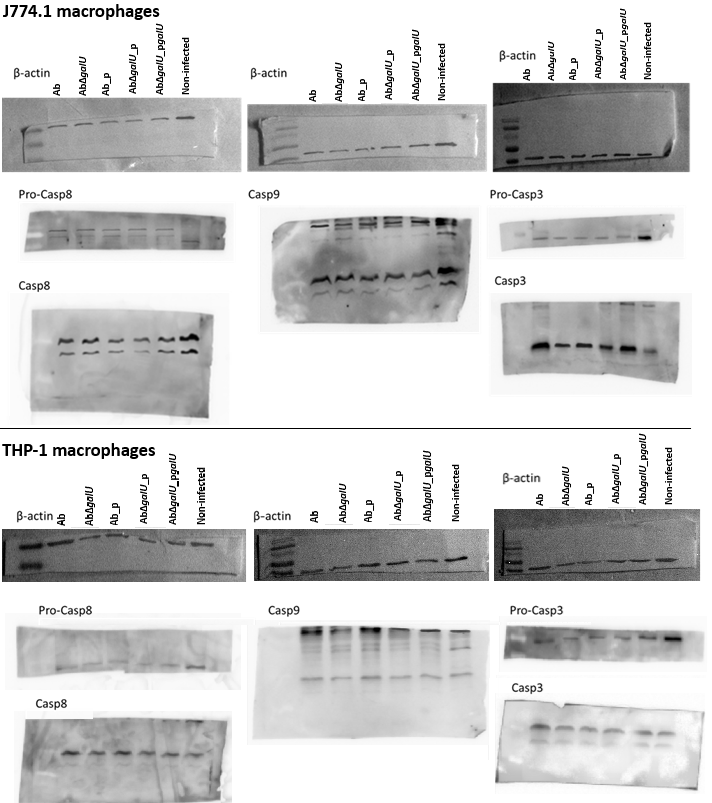
**

**
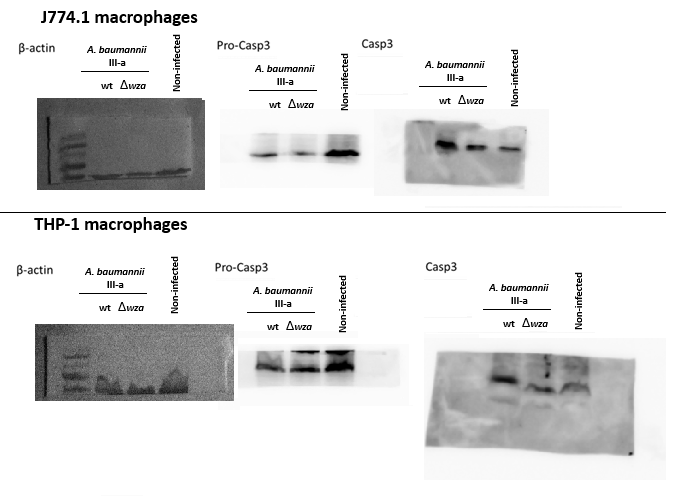
**

**Supplementary Figure S6.** Images of the original blots.
